# Insights on rehabilitation programs, women, families, and COVID19

**DOI:** 10.1038/s41398-022-02008-7

**Published:** 2022-06-09

**Authors:** Sivan Kinreich

**Affiliations:** Department of Psychiatry, State University of New York Downstate Medical Center, Brooklyn, NY USA

**Keywords:** Addiction, Scientific community

Dear Editor,

Rehabilitation programs for people suffering from alcohol and other drug use disorders are challenged with low rates of participants and high rates of relapse according to the 2019 National Survey on Drug Use and Health, USA (NSDUH). In addition, the COVID19 pandemic has added new complexity to the participation in rehabilitation programs, such as the need for quarantine and restrictions on indoor gatherings. A reassessment of the structure of rehabilitation programs to evolve into a system that encourages individuals, especially women, with varying personal circumstances to enter and be maintained in treatment, is of utmost importance.

The availability of rehabilitation programs in the USA has substantially increased since 2008 when two laws were passed. The first law is The Mental Health Parity and Addiction Equity Act (MHPAEA), which was passed in 2008, and the second law, passed in 2010, named The Affordable Care Act (ACA). However, even years later, only 12.2% of people aged 12 or older who needed substance use treatment received it in the USA. These estimates in 2019 were similar to the estimates in each year from 2015 to 2018 (NSDUH, 2019).

There are several treatment options for people suffering from alcohol or other drug use disorders; long-term residential treatment, short-term residential treatment, and outpatient treatment programs. Various reasons have been suggested to explain the low participation rates in these programs, including treatment-related stigma, lack of motivation for change, and limited knowledge about the value of therapy [1].

Findings from our recent paper in *Translational Psychiatry* propose looking at “treatment avoidance” (addicted people who think they need treatment but do not participate in rehab programs) from a different perspective [2]. In this machine learning study, a predictive model of remission from alcohol use disorder was built, with multimodal features (biological markers and phenotypes), and sex and ancestry stratified analysis [2]. One of the findings was that European American (EA) women and African American (AA) men who were not married and AA men who were not employed, were more likely to be in remission from AUD in the next few years (compared to those who were married and employed).

Results may be attributed to the difficulty of married (with children) and employed individuals being absent for long periods of time, as opposed to unmarried and non-working individuals [3]. The first crucial step for recovery requires leaving for weeks/months to a residential setting. This disengagement may raise stress and concerns related to job loss, and family care [3]. Especially mothers may be apprehensive about caring for their children emotionally and financially while they are in treatment [4]. These concerns can impel married and employed individuals to postpone and avoid treatment opportunities and possible recovery.

Previous studies based on nationwide surveys that reviewed the reasons for avoiding treatment have not found marital status/family/children-care nor employment variables as leading causes [5]. However, 12 and older was the age range in NSDUH and 18 and older in the National Epidemiologic Survey on Alcohol and Related Conditions (NESARC), and the details about avoiding treatment were not stratified by age or family status. In fact, the survey did not list family care as one of the reasons for not entering treatment (NSDUH, 2019). Moreso, studies that focused on sex differences in barriers to treatment entry emphasized that women reported responsibilities for children coupled with lack of childcare outside of treatment as a barrier [6, 7]. In this regard, it was found that women prefer AUD treatment settings that offer childcare [6]. Concerns about the family’s economy while in treatment with a focus on individuals with children, were also lacking in the national surveys (NSDUH, 2019).

Even if data were available and studies considered age/family/employment status in their analysis, it would not be surprising that due to the nature of the disorder, the first reasons for refraining from treatment would be “not being ready to stop using” (39.9%) or “not knowing where to go for treatment” (23.8%) (NSDUH, 2019). However, we argue that in an attempt to make rehabilitation treatments accessible, the needs of the family should be researched and translated into new treatment approaches (such as “stay-at-home rehabilitation” (SHR)).

Previous studies have indicated the many benefits of disconnecting the addicted individual from his/her known/triggering environment to the rehabilitation facility [8]. To address this and other conceptual cornerstones of the rehab process, the new programs, such as SHR, will need to include new ideas and innovative approaches. For example, one of the foremost challenges in the home recovery approach is the first stage of detox from the physiological addiction. The medical recommendation is not to attempt to detox from alcohol alone [9], thus the new programs will need to ensure a safe environment for this step.

The next steps may include online treatments as it has been shown to be effective and reliable during the recent COVID19 epidemic [10]. Rehabilitation programs intertwined with day-to-day life will manifest what was previously unattainable and thus have the potential to attract larger populations, including women and their distinctive needs [11], employed functioning individuals, people living in rural areas, as well as reducing federal and state costs. This approach is particularly relevant in the current era of the COVID19 epidemic, where people are sometimes forced to stay at home for long periods.

Sixteen million people aged 12 or older in 2019 were heavy alcohol users in the USA (NSDUH, 2019). Alcohol addiction was, and still is, one of the more complex challenges for our economic and health system. New approaches and out-of-the-box thinking should be taken in order to increase participation in rehabilitation programs.
